# Tongue Strength and Endurance in Patients with Parkinson’s Disease: Association with Swallowing, Oral Phase Efficiency, Meal, Diet Type and Malnutrition Risk

**DOI:** 10.1007/s00455-025-10902-7

**Published:** 2025-11-03

**Authors:** Francesco Mozzanica, Nicole Pizzorni, Daniela Ginocchio, Sarah Feroldi, Federica Bianchi, Micol Castellari, Gabriele Mora, Marco Gitto, Federico Ambrogi, Antonio Schindler

**Affiliations:** 1 Department of Clinical Sciences and Community Health, Università degli Studi di Milano, Milan, Italy; 2 Department of Otorhinolaryngology, IRCCS Multimedica, Milan, Italy; 3 Phoniatric Unit, Department of Biomedical and Clinical Sciences, Università degli Studi di Milano, Milan, Italy; 4 ALS Center, IRCCS Istituti Clinici Scientifici Maugeri, Milan, Italy

**Keywords:** Parkinson disease, Deglutition disorders, Tongue, Malnutrition

## Abstract

Tongue motor impairment has been documented and associated with the severity of dysphagia in patients with Parkinson’s disease (PD). Yet, no study investigates the relation between tongue measures and oral phase, swallowing performance during meal, and nutrition in this population. The study aims to measure maximum isometric tongue pressure (MIP) and endurance in patients with PD and to study their association with swallowing- and meal-related safety and efficiency, oral phase efficiency, diet type, and malnutrition risk. Thirty-tree patients with PD were enrolled. Tongue MIP and endurance were measured using the Iowa Oral Performance Instrument. Patients underwent a Fiberoptic Endoscopic Evaluation of Swallowing (FEES). Meal safety and efficiency were evaluated with the Mealtime Assessment Scale (MAS), while the Test of Masticating and Swallowing Solids (TOMASS) was used to analyze oral phase efficiency. Diet type was described according to the Functional Oral Intake Scale (FOIS) and malnutrition risk was assessed using the Mini Nutritional Assessment (MNA). The median MIP was 40 kPa, while the median tongue endurance was 14 s. At univariate regression analysis, both MIP and tongue endurance were significantly (*p* < 0.05) associated with TOMASS, MAS, FOIS, and MNA, while a significant association with FEES was only found between MIP and the severity of residue in the pyriform sinus with liquids. In conclusion, reduced tongue strength and endurance seem to be associated with the worsening of oral phase efficiency, swallowing performance during meal, diet type and malnutrition risk, but not with pharyngeal signs of dysphagia in PD.

## Introduction

Parkinson’s disease (PD) is a chronic progressive neurodegenerative disorder with an estimated prevalence of 6.1 million individuals globally [1]. It is characterized by several motor features, such as tremor, bradykinesia, and rigidity, as well as non-motor features, such as autonomic dysfunction, sleep disorders, cognitive impairment, depression, and psychosis, all of which can impact functioning to a variable degree. The clinical presentation may be heterogeneous, since these features may occur at any time during the disease course, although become more frequent with advanced disease [2].

Dysphagia is extremely common in patients with PD with a prevalence ranging between 40 and 80% depending on the disease stage, disease duration, and type of assessment performed [3]. It may limit the safe ingestion of adequate amounts of food and liquids, thus increasing the risk for malnutrition and dehydration. In addition, it can negatively affect the quality of life of individuals due to the progressive difficulties experienced at mealtimes and the limitation of eating-related social activities [4]. Moreover, affected patients may experience an increased risk of hospital admission, delayed discharge, dependence on health services, severe complications and even death [5].

All phases of swallowing phases may be affected in patients with PD [6], but the exact pathophysiological mechanism which leads to dysphagia is yet to be understood [7]. Given that the tongue plays a pivotal role in swallowing by contributing to the bolus formation, placement, and transport within the oral cavity, while also generating the driving force for bolus propulsion through the pharynx and into the upper esophageal sphincter [8, 9], tongue motor impairment has been largely studied in this population [10, 11] and movement abnormalities during both the oral and pharyngeal phases of swallowing have been demonstrated [12–14]. In patients with PD, the tongue has difficulty forming, controlling, and propelling the bolus [15], it performs repetitive forward and backward motions (“tongue pumping”) [16], and has a reduced endurance and anterior tongue strength [10].

Tongue strength was found to reduce with disease progression. Pitts et al. [10] in their systematic review found a moderate inverse correlation between tongue strength and Hoehn and Yahr staging with significant reductions in anterior tongue strength occurring between stage 1 to stage 2.5 or higher. Nevertheless, as there is no clear association between dysphagia onset and disease stage [17], further investigation on the relationship between tongue pressured and disease severity are required.

Significant associations between tongue strength and dysphagia were demonstrated. Minagi et al. [7] evaluated the tongue pressure on the hard palate while swallowing 5 ml of water in 30 patients with PD and found that the tongue pressures during swallowing were significantly lower in patients with dysphagia compared to those without dysphagia. Similarly, Fukuoka et al. [12] analyzed tongue pressure in 24 patients with PD who underwent a videofluoroscopic swallowing study (VFSS) and concluded that non-dysphagic and dysphagic PD patients demonstrated significant differences in the temporal aspects of tongue pressure. Tongue strength was significantly associated with the amount of residue in the hypopharynx [13] and the occurrence of penetration or aspiration [14] with liquids. However, information concerning the relationships between tongue measures and oral phase efficiency, swallowing performance during meal, diet type, and nutritional status is scarce.

Understanding the association between tongue pressure and endurance and the above-mentioned factors, may shed light on the role of the tongue in determining the severity of mealtime difficulties in patients with PD, thus providing information to guide its management in this population. Indeed, although instrumental assessments of swallowing are the “gold standard” for dysphagia diagnosis, they allow to evaluate only a limited number of swallows and use standard foods. Therefore, the safety and the efficiency of swallowing assessed during fiberoptic endoscopic examination of swallowing (FEES) or VFSS may not always overlap the safety and the efficiency of swallowing during a whole meal. In fact, personal and environmental factors, as well as functions other than swallowing, such as food characteristics, appetite, cognitive status, or fatigue, may impact swallowing performance in everyday life.

The aims of this study are: (1) to measure the tongue strength and endurance in patients with PD, and (2) to analyze their association with swallowing safety and efficiency assessed through instrumental swallowing examination and during meal, oral phase efficiency, diet type, and malnutrition risk. The relevance of this study lies in the fact that a deeper understanding of the characteristics of tongue strength and endurance impairment in patients with PD may prove to be useful in clinical practice and allow for improved meal management.

## Materials and Methods

This study was approved by the institutional review board (n.2016/ST/262). All procedures performed in the study are in accordance with the 1964 Helsinki declaration and its later amendments or comparable ethical standards. All patients provided written informed consent to participate in the study.

### Participants

Patients were consecutively recruited among inpatients and outpatients of one neurological center in Italy over a 30-month period. Inclusion criteria were: age ≥ 18 years; confirmed diagnosis of PD by a neurologist specializing in movement disorders; full oral diet with at least one consistency; functional oral status (all natural teeth or partial tooth loss rehabilitated with an adjusted partial dental prosthesis [18]); no medical history of gastroenterological, respiratory, rheumatologic, metabolic, hematologic disorders. Exclusion criteria were: intolerance to the components of the tested foods; additional neurologic diseases; history of head and neck cancer; previous history or concurrent involvement of other diseases affecting swallowing function; changes in anti-Parkinson’s medications or dosages within 2 weeks prior to participation.

Age, sex, disease duration, history of aspiration pneumonia, use of nutritional supplements, and disease severity (rated by a neurologist using the modified Hoehn&Yahr [19] scale) were collected. The Functional Oral Intake Scale (FOIS) [20–22], a seven-point ordinal scale indicating limitations in oral feeding which ranges from one (nothing by mouth) to seven (total oral diet with no restriction), was used to collect information regarding patient’s oral intake. The FOIS was rated immediately before the clinical examination by the same SLP that conducted meal observation and administered the TOMASS based on the patient’s typical oral intake.

Each enrolled patient underwent a multidimensional assessment, including tongue strength and endurance measures, swallowing- and meal-related safety and efficiency, oral phase efficiency, and malnutrition risk. In case of ongoing pharmacological treatment for PD, patients were evaluated during their best on-state, approximately 30–45 min after taking PD medication.

### Tongue Strength and Endurance

To objectively measure tongue strength and endurance the Iowa Oral Performance Instrument (IOPI) (model 2.3; IOPI Medical LLC, Carnation, WA) was used [23]. This is a portable device which measures the pressure (kPa) exerted by the tongue on an air-filled pliable plastic bulb attached to a pressure transducer. For the tongue strength measurement, the patients were seated in an upright position and instructed to place the bulb between the midline of the tongue and the hard palate, directly behind the upper alveolar ridge. To ensure the same positioning among different trials, a mark was made on the tube of the bulb immediately anterior to the incisors. Patients were instructed to press against the tongue bulb using as much effort as possible. At least 3 trials were collected. Patients were given a 1-min rest period between trials. The maximum isometric tongue pressure (MIP) was defined as the highest of the three peak isometric tongue pressure scores. The MIP was considered a reflection of the patient’s maximum tongue blade strength in an upward direction [8].

Tongue endurance was defined as the maximum time the patients were able to sustain 50% of their MIP. The target pressure was calculated and set using the arrows on the device (until the desired pressure was indicated on the display). The bulb was positioned identically to the MIP evaluation and the patients were instructed to sustain the target pressure for as long as possible. A series of LED lights on the right side of the device automatically alerted the patients when they reached the target pressure. Time was measured using the stopwatch incorporated in the IOPI device. Timing started when the pressure meets the target pressure and stops when the pressure drops steeply, the pressure is maintained between 40% and 50% of MTP for 2 s or more, or the pressure stays below 40% of MTP for at least 0.5 s [9]. The endurance measurement was repeated 3 times, with a resting period of 2 min between the trials. For the purpose of this study, the best of the three attempts, measured in seconds, was considered. All measures were acquired on the same day as the FEES, at least 1 h after meals and before FEES to avoid potential fatigue effects secondary to meal consumption or FEES trials.

### Swallowing Safety and Efficiency Assessment

The swallowing assessment was performed by a phoniatrician with >10 years of experience in FEES. A XION EF-N flexible endoscope with a diameter of 3.4 mm and a length of 320 mm (XION GmbH, Berlin, Germany) mounted on an EndoSTROBE camera (XION GmbH, Berlin, Germany) was used. All the videos were stored in an anonymized form. Each patient was seated on a comfortable chair, leaning back (75–90° approximately) with his arms on the armrests and maintaining a neutral head position to obtain the best posture for the examination. No local anesthetic drugs were used in order not to alter pharyngo-laryngeal sensibility. The endoscope was introduced into the widest nasal cavity and positioned directly below the uvula to maximize the field of view, including the larynx, the valleculae and the pyriform sinuses. Three different food consistencies were provided during FEES examination to evaluate swallowing: liquids (5–10–20 ml of room temperature blue-died water ×3 trials for each volume; International Dysphagia Diet Standardization Initiative [IDDSI] = 0 [24]; <50 mPa·s at 50 s-1 and 300 s-1), semisolids (5–10–20 ml of room temperature Crème Line vanilla pudding ×3 trials for each volume; IDDSI = 4; 2583.3 ± 10.41 mPa·s at 50 s-1 and 697.87 ± 7.84 mPa·s at 300 s-1), and solids (half biscuit of an 8-gram dry biscuit ×2 trials; IDDSI = 7).

The protocol was reduced if a consistency or a volume was not considered safe to be administered according to the following criteria: (a) a Penetration-Aspiration Scale (PAS) score [25] ≥ 7 was recorded on smaller volumes of the same consistency or on previous instrumental assessments, (b) the patient was not able to hold the bolus in the mouth because of anterior leakage from the lips, or (c) mastication inability or severe difficulties in oral transport of the bolus into the pharynx (this criteria applied only to solids).

FEES examinations were rated independently by two operators using the video files. Both were speech and language therapists (SLTs) with at least 5 years of experience in FEES examinations. SLTs were blind to the other’s evaluation and to the participants’ data, since videos were stored in anonymized form. The safety and efficiency of swallowing were rated using validated ordinal scales. In the case of an inter-rater difference > 1 level at any FEES rating scale, a 3rd SLT assessed the videos.

For the swallowing safety evaluation, the PAS was used [25]. This is an 8-points scale ranging from 1 (materials do not enter the airway) to 8 (materials enter the airway, passes below the vocal folds and no effort is made to eject). Penetration is defined as the bolus entering the laryngeal vestibule over the rim of the larynx (PAS 2–5). Aspiration is defined as the bolus passing below the true vocal folds (PAS ≥ 6). On the other hand, the efficiency of swallowing was evaluated using the Yale Pharyngeal Residue Severity Rating Scale (YPRSRS) vallecula and pyriform sinus [26, 27]. This scale rates the amount of residue in the valleculae and pyriform sinuses after the swallow. It ranges from 1 to 5, with higher scores indicating a larger amount of residue after swallow.

### Meal Safety and Efficiency Assessment

Meal observation was performed to evaluate swallowing performance during meal. Patients were observed by an SLT while consuming a typical meal. Swallowing safety and efficiency during meal consumption were assessed using the Mealtime Assessment Scale (MAS) [28, 29]. The MAS provides a meal safety score (range 0–12) and a meal efficiency score (range 0–18) based on the signs of dysphagia detected during meal. The higher the score, the less safe or efficient the swallowing function is during the meal. Additionally, the protocol requires the evaluator to indicate the time needed to complete the meal.

### Oral Phase Efficiency Assessment

The mastication assessment was performed through the internationally validated Test of Masticating and Swallowing Solids (TOMASS) [30, 31]. The test requires ingestion of a standard GranPavesi cracker with the instructions to eat it “as quickly as is comfortably possible”. The quantified measures include the number of discrete bites, number of masticatory cycles per bite, number of swallows per bite, and total time. The TOMASS was video-recorded, anonymized, and assessed by 2 independent SLTs.

### Risk of Malnutrition

The risk of malnutrition was assessed using the Mini Nutritional Assessment (MNA) [32]. This tool consists of eighteen questions divided into four domains which include diverse components, such as: weight loss and depletion in food access in the past 3 months, changes in physical activity, psychological stress, and acute illness in the mentioned period, as well as anthropometric measurements, altered sense of smell and taste, or frailty. Patients scoring 17–23.5.5 are considered at risk of malnutrition. Patients with a score < 17 are considered malnourished.

### Statistical Analysis

Statistical analysis was performed with IBM SPSS Statistics v28.0 and R free software (R Core Team, 2023, <https://www.R-project.org/%3E). Kruskal-Wallis and Mann-Whitney tests were used to compare the distribution of tongue strength and endurance among the different stages of PD (as assessed using the Hoehn&Yahr scale) and between males and females. Univariate linear regression was performed to estimate the degree of association between tongue strength and endurance (independent variables) and swallowing, mastication, meal and nutritional variables (dependent variables). Other independent variables were age, gender, disease duration, disease severity, and type of oral intake. Possible mild non-linear effects were considered using restricted cubic splines with 3 knots (placed at 0.1, 0.5 and 0.9 percentiles). When non-linear effects were non-significant only the linear component was fitted. The association of continuous variables with the outcomes is reported by quantifying the change in outcome for a change from the first to the third quartile of each continuous independent variable. Considering the sample size, no multivariable analysis was performed. Significance was set at *p* < 0.05. Missing values were excluded on a pairwise basis.

### Sample Size

The study was part of a larger study on the association between dysphagia and nutritional risk in neurodegenerative diseases. A sample size calculation of 47 patients was performed, based on expected association with swallowing and malnutrition risk equal to 0.4 (alpha = 0.05 and Beta = 0.20). Due to limited access to FEES, 33 patients with PD were recruited. This sample size had the power to detect a correlation coefficient equal to 0.49.

## Results

A total of 33 patients with a confirmed diagnosis of PD were recruited (25 males and 8 females) with a median age of 77 years (range 45–84, 75% of the patients were aged ≥ 70). Characteristics of the recruited population are reported in Table 1. None of the enrolled patients had a history of aspiration pneumonia or used nutritional supplements. Median disease duration (years from diagnosis) was 9 years (range 2–28 years). The Hoehn&Yahr stage was 1.5 in 5 patients, stage 2 in 4 patients, stage 2.5 in 6 patients, stage 3 in 9 patients, stage 4 in 8 patients, and stage 5 in 1 patient. With regard to diet type, 3 patients had a total oral diet with a single consistency (FOIS = 4), 3 patients had a total oral diet with multiple consistencies requiring special preparations or compensations (FOIS = 5), 20 patients had a total oral diet with multiple consistencies without special preparation but with specific food restrictions (FOIS = 6), and the remaining 8 patients had a total oral diet with no restrictions (FOIS = 7).

**Table 1:** Characteristics of the study sample

Characteristics of the enrolled cohort	
Gender	25 males 8 females
Age	72, 77 years (70–78 years)
Disease duration	9, 9 years (4–12 years)
Modified Hoehn & Yahr score	2.9, 3 (2–4)
FOIS	6, 6 (6–6)
BMI	25.0, 24.7 (22.0–27.3.0.3)

Note. The mean (first) and median (second) scores are reported as well as the interquartile range (in brackets)

LEGEND. BMI = body mass index; FOIS = Functional Oral Intake Scale

### Tongue Strength and Endurance

**Fig. 1:**
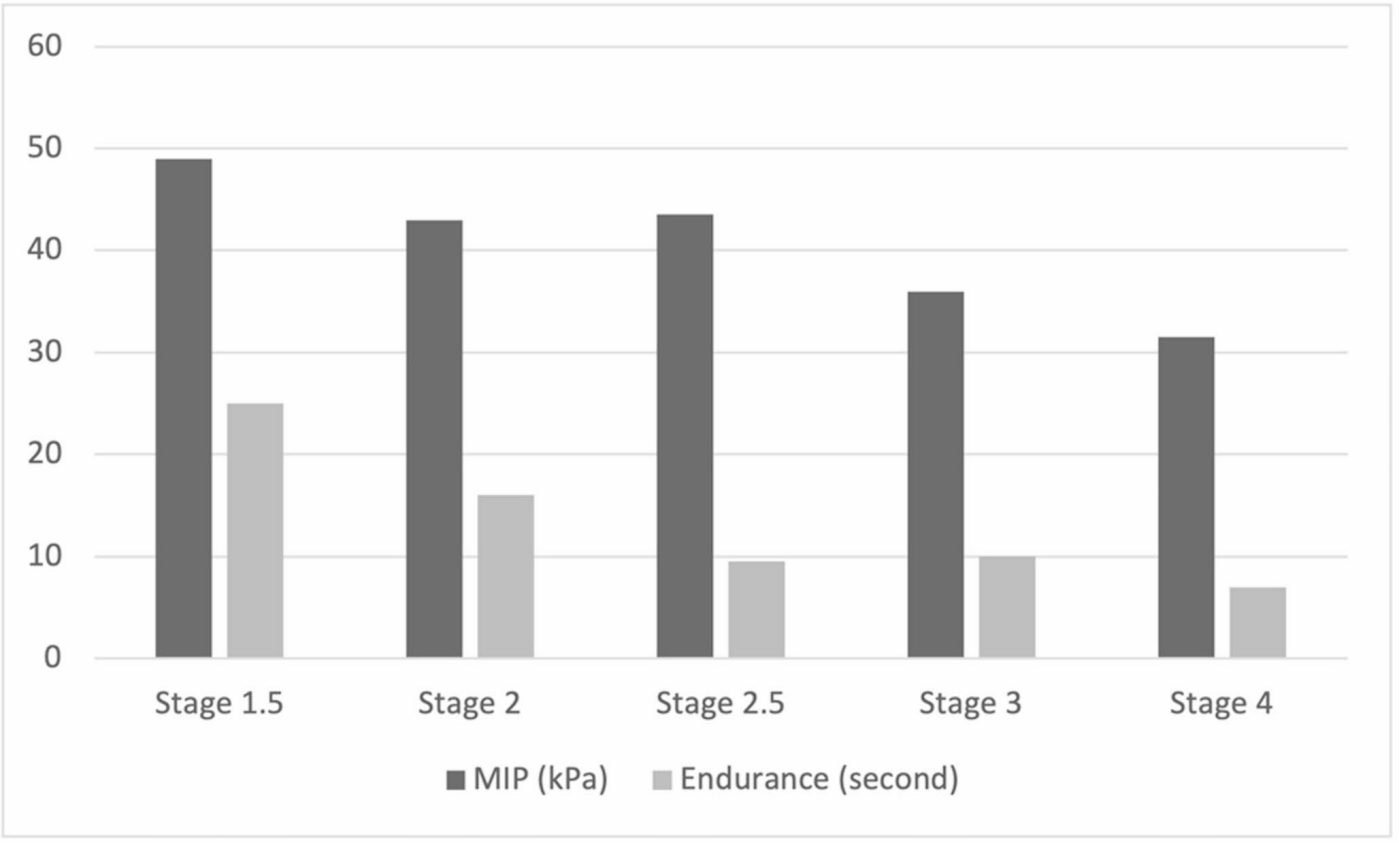
distribution of MIP (maximum isometric tongue pressure in kPA) and tongue endurance (in seconds) according to the modified Hoen & Yahr stage of Parkinson’s disease

All the enrolled patients were able to perform the IOPI evaluation. The median MIP score was 40 kPa (IQR 32–51 kPa), while the median tongue endurance was 14 s (IQR 6–25 s). No differences in the distribution of MIP and endurance were demonstrated between males and females (MIP females median 40 kPa [IQR 26.75–50.5], males median 40 kPa [IQR 32–52], *p* = 0.911; endurance females median 9.5 s [IQR 6.5–16], males median 15 s [IQR 5–27], *p* = 0.758 at Mann-Whitney U test) nor among the different stages of PD according to the modified Hoehn & Yahr scale (*p* = 0.457 and *p* = 0.202 respectively at Kruskal-Wallis test) (Fig. 1).

### Swallowing Assessment

**Fig. 2:**
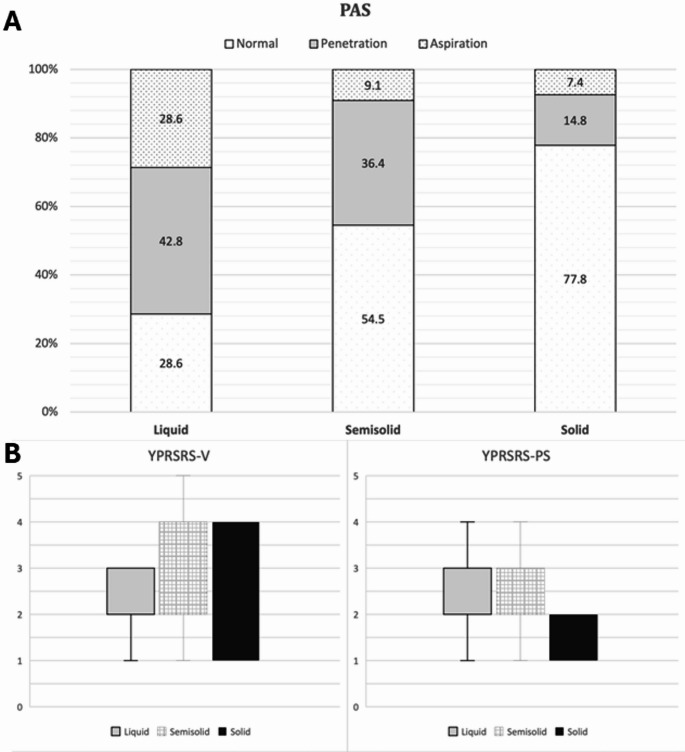
Swallowing safety (A) and efficiency (B) according to FEES examination Results of the PAS scores in the enrolled patients. The PAS scores were categorized in Normal, Penetration (PAS score from 2 to 5) and Aspiration (PAS score 6 or above). PAS = Penetration Aspiration Scale Box plots reporting the efficiency of swallow analyzed through the YPRSRS. YPRSRS-V = Yale Pharyngeal Residue Severity Rating Scale Vallecula; YPRSRS-PS = Yale Pharyngeal Residue Severity Rating Scale Pyriform Sinuses

The swallowing assessment was performed for all the enrolled patients and the examination was well tolerated. FEES protocol was performed using all the three consistencies in 27 patients. Solid was not tested in 6 patients, liquid was not tested in 5 patients, while semisolid was always tested. The time required to complete FEES never exceeded 10 min. The results of the PAS and YPRRS vallecula and pyriform sinus are reported in Fig. 2. Interestingly, aspiration and/or penetration were present in 71.4% of the patients with the liquid consistency, in 45.5% with the semisolid texture, and in 22.2% with the solid texture (Fig. 2a). As far as the efficiency of swallow is concerned, at least mild residue (YPRRS ≥ 3) in the valleculae were found in the 71.4% (20 out of 28) of the patients with the liquid texture, 69.7% (23 out of 33) with the semisolid texture, and 62.9% (17 out of 27) with the solid texture. At least mild residue in the pyriform sinuses were found in 53.6% (15 out of 28) of the patients with the liquid texture, 42.4% (14 out of 33) with the semisolid texture, and 22.2% (6 out of 27) with the solid texture (Fig. 2b).

### Meal Safety and Efficiency Assessment

Meal observation was performed in all the enrolled patients and the results of the MAS are reported in Table 2. The median meal safety and efficiency scores were 2 and 1 respectively, while the median time required to complete a typical meal was 20 min. No differences in the distribution of MAS scores between males and females and among patients with different grade of PD were demonstrated at non-parametric tests.

**Table 2:** Results of the MAS and TOMASS

Scale column	Variable column	Score
MAS	Meal safety	2 (1–4)
	Meal efficiency	1 (0–2)
	Time (minutes)	20 (15–25)
TOMASS	Bites	2 (1–3)
	Masticatory	43 (31–63)
	Swallow	2 (2–3)
	Time (seconds)	37 (32–52)

Note. Median and interquartile ranges are reported. Bites: number of discrete bites; Masticatory: number of masticatory cycles per bite; Swallow: number of swallows per bite

### Oral Phase Efficiency Assessment

TOMASS was administered in 27 patients (the remaining 6 were considered at risk of choking at the FEES examination with this consistency, therefore it was not tested). The results are reported in Table 2. Measures above normative data were recorded in 10 patients (30.3%) for time, 8 (24.2%) patients for bites and masticatory cycles, and in 3 patients (9.1%) for swallows. No differences in the distribution of TOMASS scores between males and females was demonstrated at Mann-Whitney test (*p* = 0.998 for “number of discrete bites”, *p* = 0.879 for “number of masticatory cycles per bite”; *p* = 0.406 for “number of swallows per bite”, and *p* = 0.931 for “total time”). Similarly, no differences in any of the TOMASS scores were demonstrated at Kruskal-Wallis test among patients with different stage of disease (*p* = 0.197 for “number of discrete bites”, *p* = 0.461 for “number of masticatory cycles per bite”; *p* = 726 for “number of swallows per bite”, and *p* = 0.815 for “total time”).

### Risk of Malnutrition

A total of 19 out of 34 patients were considered at risk of malnutrition and 2 patients were considered malnourished. The MNA median score was 22 (interquartile range 19.5–27).

### Regression Analysis

**Fig. 3:**
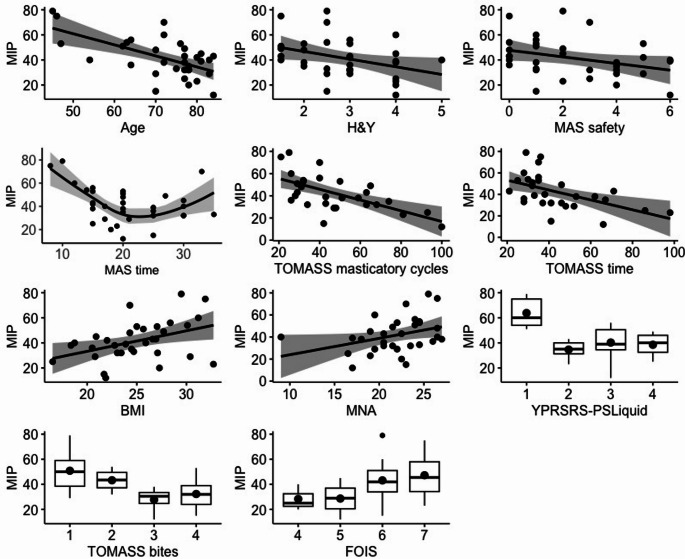
To fully evaluate the association between MIP (maximum isometric tongue pressure in kPA) with swallowing and meal safety and efficiency, oral phase efficiency, diet type, and malnutrition risk through regression analysis, the association with significant factors is reported in the figure. For continuous predictors the regression line is shown, either a straight line or a non-linear relationship when spline is used. For categorical predictors the average predicted MIP (with SE) is reported for each level

**Fig. 4:**
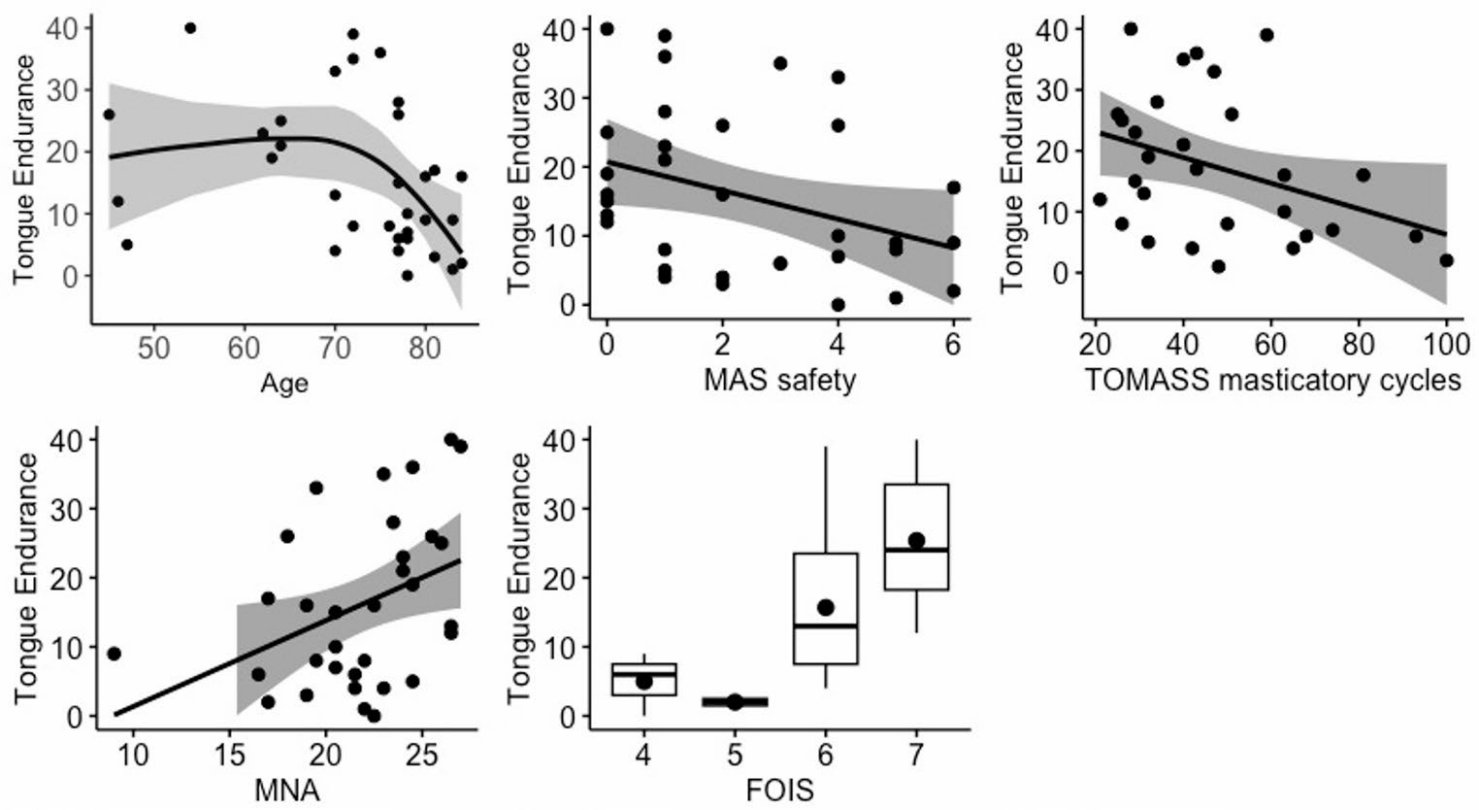
To fully evaluate the association between tongue endurance (in seconds) with swallowing and meal safety and efficiency, oral phase efficiency, diet type, and malnutrition risk through regression analysis, the association with significant factors is reported in the figure. For continuous predictors the regression line is shown, either a straight line or a non-linear relationship when spline is used. For categorical predictors the average predicted MIP (with SE) is reported for each level

The results of the regression analysis are reported in Tables 3 and 4 and in Figs. 3 and 4. Considering the associations with MIP, a significant negative association was found with age (MIP decrease by 7.07 by increasing age from 70 to 78 years), modified Hoehn & Yahr scale score (MIP decrease by 12.2 by increasing the score from 2 to 4), MAS time (MIP decrease by 15.41 by increasing MAS time from 15 to 25), MAS safety (MIP decrease by 7.93 by increasing MAS safety from 1 to 4), TOMASS number of masticatory cycles per bites (MIP decrease by 15.50 by increasing TOMASS number of masticatory cycles from 31 to 63), TOMASS total time (MIP decrease by 9.18 by increasing TOMASS total time from 32 to 52). Alternatively, MIP was positively associated with BMI (MIP increase by 7.45 by increasing BMI from 19.5 to 24.5), and MNA (MIP increase by 8.64 by increasing MNA from 22.0 to 27.3). Considering the categorical variables, a significant association was found with the amount of residue of liquid in the pyriform sinuses (considering YPRSRS score 3 – mild residue - as reference, score 1 – no residue - has a MIP value higher by 23.53, while score 2 – traces - and 4 – severe residue - are comparable), TOMASS number of discrete bites (Considering 1 bite as reference, category 2 is comparable while 3 and 4 have a lower value of MIP by about 20) and FOIS (Considering level 6 as reference, levels 4 and 5 have a lower value by about 14 while level 7 is comparable).

**Table 3:** Univariable regression analysis for tongue strength

Variable	Type of variable	β	Contrast	Effect (95% CI)	*p*
Sex	Dichotomic		F vs. M	−2.62	0.69
Age	Continue	−0.88	3Q vs. 1Q 78 vs. 70	−7.07 (−10.43; −3.71)	**< 0.01**
Disease duration	Continue	2.15 −3.07	3Q vs. 1Q 12 vs. 4	6.55 (−3.60;16.70)	0.08
H&Y	Continue	−6.12	3Q vs. 1Q 4 vs. 2	−12.2 (−23.70; −0.80)	**0.04**
PAS Liquid	Continue	−1.11	3Q vs. 1Q 7 vs. 2	−5.55 (−17.31;6.21)	0.34
PAS Semisolid	Continue	−1.44	3Q vs. 1Q 3 vs. 1	−2.88 (−8.85;3.09)	0.33
PAS Solid	Continue	−0.18	3Q vs. 1Q 1.5 vs. 1	−0.09 (−1.83;1.65)	0.92
YPRSRS-V Liquid	Categorical (1,2,3)		1 vs. 3 2 vs. 3	21.38 (4.1;38.6) 2.05 (−11.9;15.9)	0.06
YPRSRS-V Semisolid	Categorical (1,2,3,4,5)		1 vs. 4 2 vs. 4 3 vs. 4 5 vs. 4	−9.58 (−44.2;24.9) 8.42 (−6.2;23.1) 3.32 (−10.9;17.5) −3.58 (−38.1;30.9)	0.70
YPRSRS-V Solid	Categorical (1,2,3,4,5)		2 vs. 1 3 vs. 1 4 vs. 1 5 vs. 1	10.5 (−9.4;30.4) −0.79 (−17.6;15.9) −0.07 (−16.9;16.7) −6.5 (−40.9; 27.9)	0.76
YPRSRS-PS Liquid	Categorical (1,2,3,4)		1 vs. 3 2 vs. 3 4 vs. 3	23.53 (11.3;35.7) −5.65 (−16.2; 4.9) −1.77 (−14.9; 11.4)	**< 0.01**
YPRSRS-PS Semisolid	Categorical (1,2,3,4)		1 vs. 2 3 vs. 2 4 vs. 2	6.69 (−9.5;22.9) −3.23 (−16.1; 9.6) 7.69 (−26.4; 41.8)	0.62
YPRSRS-PS Solid	Categorical (1,2,3,4)		2 vs. 1 3 vs. 1 4 vs. 1	8.8 (−5.8;23.4) 10.5 (−5.1;26.2) 22.1 (−9.1;53.4)	0.265
MAS safety	Continue	−2.64	3Q vs. 1Q 4 vs. 1	−7.93 (−16.02;−0.16)	**0.049**
MAS efficiency	Continue	0.32	3Q vs. 1Q 2 vs. 0	0.64 (−8.22;9.49)	0.89
MAS time	Continue	−3.53 5.52	3Q vs. 1Q 25 vs. 15	−15.41 (−23.29;−7.53)	**< 0.01**
TOMASS bites	Categorical (1,2,3,≥4)		2 vs. 1 3 vs. 1 4 vs. 1	−7.61 (−23.9;8.7) −23.11 (−39.4;−6.8) −18.57 (−31.9;−5.3)	**0.01**
TOMASS masticatory cycles	Continue	−0.48	3Q vs. 1Q 63 vs. 31	−15.50 (−23.18; −7.82)	**< 0.01**
TOMASS swallows	Dichotomic (0 = normal; 1 = outside normative data)		1 vs. 0	−10.56 (−30.91;9.79)	0.30
TOMASS time	Continue	−0.46	3Q vs. 1Q 52 vs. 32	−9.18 (−15.02; −3.33)	**< 0.01**
FOIS	Categorical (4,5,6,7)		4 vs. 6 5 vs. 6 7 vs. 6	−14.83 (−34.0;−4.3) −14.49 (−33.7;−4.7) 4.09 (−8.9;17.1)	**0.04**
MNA	Continue	1.49	3Q vs. 1Q 24.5 vs. 19.5	7.45 (0.11;14.80)	**0.047**
BMI	Continue	1.64	3Q vs. 1Q 27.3 vs. 22	8.64 (1.73; 15.55)	**0.02**

Note. For continuous covariates a regression with a restricted cubic spline with 3 knots (placed at 0.1, 0.5 and 0.9 percentiles) was used to account for possible mild nonlinear effects. In case of non-significant nonlinear effect, only the linear one was estimated. Therefore, the table reports one regression coefficient in case of non-significant nonlinear effect for continuous covariates, otherwise two coefficients are reported (**β**). As it is difficult to guess the association of covariates when splines are fitted reporting two coefficients, to evaluate the degree of association the predicted change (Effect) of tongue strength from the first to the third quartile of each continuous covariate is reported (Contrast). For example, considering BMI, the first quartile of BMI is 22, while the third quartile is 27.3. The average difference in tongue strength for patients with BMI 22 with respect to patients with BMI 27.3 is equal to the Effect, i.e. 8.64. For categorical covariates, the contrast (Effect) between the different categories with respect to the median category is reported (Contrast)

LEGEND. H&Y = Hoehn & Yahr score; PAS = Penetration Aspiration Scale; YPRSRS-V = Yale Pharyngeal Residue Severity Rating Scale Vallecula; YPRSRS-PS Yale Pharyngeal Residue Severity Rating Scale Pyriform Sinuses; MAS = Mealtime Assessment Scale; TOMASS = Test of Masticating and Swallowing Solids; FOIS = Functional Oral Intake Scale; MNA = Mini Nutritional Assessment; BMI = Body Mass Index

**Table 4:** Univariable regression analysis for tongue endurance

Variable	Type of variable	β	Contrast	Effect (95% CI)	*p*
Sex	Dichotomic		F vs. M	−5.04	0.30
Age	Continue	0.13 −0.82	3Q vs. 1Q 78 vs. 70	−7.04 (−11.96; −2.11)	**0.02**
Disease duration	Continue	−0.39	3Q vs. 1Q 12 vs. 4	−3.15 (−8.58;2.28)	0.24
H&Y	Continue	−4.22	3Q vs. 1Q 4 vs. 2	−8.45 (−17.17; 0.27)	0.06
PAS Liquid	Continue	−1.11	3Q vs. 1Q 7 vs. 2	−5.55 (−17.31;6.21)	0.34
PAS Semisolid	Continue	0.01	3Q vs. 1Q 3 vs. 1	0.03 (−4.53;4.59)	0.99
PAS Solid	Continue	−0.36	3Q vs. 1Q 1.5 vs. 1	−0.18 (−1.54;1.18)	0.79
YPRSRS-V Liquid	Categorical (1,2,3)		1 vs. 3 2 vs. 3	8.52 (−5.8;22.9) 3.85 (−7.7;15.4)	0.43
YPRSRS-V Semisolid	Categorical (1,2,3,4,5)		1 vs. 4 2 vs. 4 3 vs. 4 5 vs. 4	−14.42 (−40.4;11.5) 2.03 (−8.9;13.0) 1.78 (−8.9;12.5) −8.42 (−34.4;17.5)	0.68
YPRSRS-V Solid	Categorical (1,2,3,4,5)		2 vs. 1 3 vs. 1 4 vs. 1 5 vs. 1	−1.13 (−16.9;14.7) 0.45 (−12.9;13.8) −3.13 (−16.5;10.3) −11.13 (−38.5;16.3)	0.91
YPRSRS-PS Liquid	Categorical (1,2,3,4)		1 vs. 3 2 vs. 3 4 vs. 3	0.15 (−11.9;12.3) 0.17 (−10.3;10.6) −12.46 (−25.6;0.7)	0.24
YPRSRS-PS Semisolid	Categorical (1,2,3,4)		1 vs. 2 3 vs. 2 4 vs. 2	−1.58 (−13.7;10.6) 3.46 (−6.2;13.1) −11.08 (−36.7;14.5)	0.60
YPRSRS-PS Solid	Categorical (1,2,3,4)		2 vs. 1 3 vs. 1 4 vs. 1	−5.57 (−17.6;6.5) 0.40 (−12.5;13.3) −10.40 (−36.2;15.4)	0.67
MAS safety	Continue	−2.07	3Q vs. 1Q 4 vs. 1	−6.22 (−12.27;−0.17)	**0.04**
MAS efficiency	Continue	−1.08	3Q vs. 1Q 2 vs. 0	−2.18 (−8.79;4.44)	0.51
MAS time	Continue	0.02	3Q vs. 1Q 25 vs. 15	0.21 (−6.24;−6.65)	0.95
TOMASS bites	Categorical (1,2,3,≥4)		2 vs. 1 3 vs. 1 4 vs. 1	−7.32 (−20.3;5.7) −4.57 (−17.6;8.4) −13.07 (−23.7;−2.5)	0.11
TOMASS masticatory cycles	Continue	−0.21	3Q vs. 1Q 63 vs. 31	−6.74 (−13.35; −0.13)	**0.046**
TOMASS swallows	Dichotomic (0 = normal; 1 = outside normative data)		1 vs. 0	−4.77 (−19.72;10.18)	0.52
TOMASS time	Continue	−0.12	3Q vs. 1Q 52 vs. 32	−2.37 (−7.27; 2.57)	0.33
FOIS	Categorical (4,5,6,7)		4 vs. 6 5 vs. 6 7 vs. 6	−10.68 (−23.2;1.9) −13.68 (−26.2;−1.1) 9.69 (1.2;18.2)	**< 0.01**
MNA	Continue	1.24	3Q vs. 1Q 24.5 vs. 19.5	6.22 (0.82;11.62)	**0.03**
BMI	Continue	−0.09	3Q vs. 1Q 27.3 vs. 22	−0.47 (−6.18; 5.24)	0.87

Note. For continuous covariates a regression with a restricted cubic spline with 3 knots (placed at 0.1, 0.5 and 0.9 percentiles) was used to account for possible mild nonlinear effects. In case of non-significant nonlinear effect, only the linear one was estimated. Therefore, the table reports one regression coefficient in case of non-significant nonlinear effect for continuous covariates, otherwise two coefficients are reported (β). As it is difficult to guess the association of covariates when splines are fitted reporting two coefficients, to evaluate the degree of association the predicted change (Effect) of tongue endurance from the first to the third quartile of each continuous covariate is reported (Contrast). For example, considering BMI, the first quartile of BMI is 22, while the third quartile is 27.3. The average difference in tongue endurance for patients with BMI 22 with respect to patients with BMI 27.3 is equal to the Effect, i.e. −0.47. For categorical covariates, the contrast (Effect) between the different categories with respect to the median category is reported (Contrast)

LEGEND. H&Y = Hoehn & Yahr score; PAS = Penetration Aspiration Scale; YPRSRS-V = Yale Pharyngeal Residue Severity Rating Scale Vallecula; YPRSRS-PS Yale Pharyngeal Residue Severity Rating Scale Pyriform Sinuses; MAS = Mealtime Assessment Scale; TOMASS = Test of Masticating and Swallowing Solids; FOIS = Functional Oral Intake Scale; MNA = Mini Nutritional Assessment; BMI = Body Mass Index

Considering the association with tongue endurance, significant negative associations were found with age (tongue endurance decrease by 7.04 by increase of age from 70 to 78), MAS safety (tongue endurance decrease by 6.22 by increase of MAS safety from 1 to 4), TOMASS number of masticatory cycles per bites (tongue endurance decreased by 6.74 when the number of masticatory cycles per bites, as evaluated through TOMASS, increased from 31 to 63), and positive association with MNA (tongue endurance increased by 6.22 when the MNA increased from 19.5 to 24.5). Considering the variables measured as categorical, a significant association was found with FOIS (considering category 6 as reference, categories 4 and 5 have a lower value by about 10 while category 7 has a higher value by about 10).

## Discussion

In the present study, tongue strength and tongue endurance were measured in patients with PD, as well as their impact on swallowing and meal safety and efficiency, oral phase efficiency, diet type, and risk of malnutrition risk. A lower tongue strength and a reduced tongue endurance were significantly associated with a reduced oral phase efficiency (TOMASS), a more impaired swallowing safety during meal (MAS), a greater malnutrition risk (MNA), and a more restricted diet (FOIS), whereas their effect on FEES parameters was minimal.

### Tongue Strength and Endurance

In our cohort, the median MIP was 40 kPa while the median endurance was 14 s. These values resemble those previously reported in patients with PD. Regarding the MIP, Pitts et al. [33] reported a mean MIP of 49 in 24 patients with PD. The same authors performed a systematic review of the literature [10] and concluded that MIP in patients with PD was significantly lower than age- and sex- matched controls, even though the reported values of tongue strength varied across the studies included (from 38.7 [34] to 55.5 [35]). Conversely, Umemoto et al. [36] reported a mean value of MIP equal to 28 in a group of 186 patients with PD, which is lower than the one found in our study. These differences might be partially related to the characteristics of the enrolled cohort. For example, in the study of Pitts et al. [33] most included patients had unrestricted or minimally restricted diets, while Umemoto et al. [36] also included patients with a diet restricted to one consistency or requiring total or partial enteral nutrition. The disease stage may have played a role since reduced MIP scores may not manifest until the later stages of PD [33]. However, the absence of information regarding the disease stage in the cohort enrolled by Umemoto et al. [36] makes it difficult to draw any conclusion on this aspect.

As far as tongue endurance is concerned, our data suggests that patients with PD experience a certain degree of tongue fatigability. This is in accordance with the reports of the systematic review of Pitts et al. [10], which concluded that tongue endurance was significantly lower in patients with PD compared to controls with approximately one-fourth of the sample scoring less than 15 s. That said, the results presented in this study appear lower than those previously reported. Solomon et al. [35] reported a mean endurance of 22.8 s in a group of 16 patients with PD, while Plaza et al. [37] found a mean value of 6.9 s in 60 patients with PD. These differences might be related to the severity of the disease of the enrolled patients. Indeed, in the study by Solomon et al. [35] only 2 out of 16 patients had a Hoehn & Yahr score higher than 3 (9/33 in the present study), while patients with such a score represented 68.4% of the cohort in the study of Plaza et al. [37].

Patients with PD did not show significant within-group differences in tongue strength or endurance when compared by sex. This is in line with the results of Plaza et al. [37]. On the other hand, the same authors demonstrated a significant difference in tongue strength in patients with different disease stages. In our cohort, even if both tongue strength and endurance seem to reduce as the severity of PD increases, we failed to detect a significant difference among different disease stages. This diverging result might be related to the relatively low sample size of the present study.

### Tongue Strength and Endurance Associations

In our study, tongue strength and endurance were found to reduce as age increases. This result is in line with the report of Plaza et al. [37], which found a significant inverse correlation between these parameters analyzed in a group of 60 patients with PD. Hoehn and Yahr stage was significantly and inversely associated with only tongue strength but not with tongue endurance. This data is in agreement with previous reports [10, 37]. In a systematic review, Pitts et al. [10] found a moderate inverse correlation between tongue strength and Hoehn and Yahr staging, but not between tongue endurance and Hoehn and Yahr stage.

As far as the results of the association analysis between FEES parameters and tongue measurements is concerned, our sample found that the only significant association was between the MIP and the amount of residue in the pyriform sinuses after a liquid swallow (lower MIP, greater pharyngeal residues). This datum is very difficult to interpret since only one previous study analyzed the association between FEES parameters and tongue strength [13]. Similar to our results, Pereida da Costa et al. [13] found that, in the presence of hypopharyngeal residue of thin liquids, the MIP was significantly associated with the amount of residue observed. In addition, the authors did not find any significant associations between MIP and penetration/aspiration events or residue with solid trials. Conversely, Sevitz et al. [14] demonstrated a significant association between MIP and PAS score with liquid that our study failed to detect. Additional research on this topic is needed to clarify this point.

Our results suggest that tongue strength and endurance significantly affect both the ability to consume a meal (assessed using the MAS) and to chew (assessed using the TOMASS). An increase in the number of masticatory cycles per bite was associated with a reduction of tongue strength and endurance, while an increase in the number of discrete bites was somewhat related to a decrease of tongue strength. Even if this association was not previously investigated in PD, the results here reported appear in accordance with the findings of Hara et al. [38] which found a significant association between tongue strength and maximum occlusal force, an indicator of chewing ability, in 785 healthy individuals. Moreover, the time to consume a cracker or a meal was found to be negatively influenced by a reduction in tongue strength. While this datum seems logical considering the role of the tongue in mastication and swallowing, it is very difficult to compare to the existing literature since, to the best of our knowledge, no previous study has analyzed the association among the above-mentioned parameters in patients with PD. However, Pitts et al. [33], who evaluated the association between tongue strength and swallowing related quality of life, found a significant association between the eating duration domain score of the Swallowing Quality of Life [39] and MIP, thus suggesting that a decrease of the tongue strength might increase the duration of the meal in patients with PD. Moreover, Sano et al. [40], who examined the relation between chewing movement and motor dysfunction in association with PD progression, found that chewing speed positively correlated with tongue pressure in patients with a mild severity PD, thus suggesting that the time required to consume a meal (or a cracker) is related to tongue strength.

Diet type and malnutrition risk, evaluated using FOIS and MNA, were found to be significantly associated with both tongue strength and endurance, while a significant relationship with BMI was demonstrated only for tongue strength. Regarding the oral intake, comparable to our results, Umemoto et al. [37] found a significant correlation between tongue strength and FOIS score in a group of 186 patients with PD. As far as the malnutrition risk is concerned, no previous study has evaluated the relationship between MNA and tongue pressures in patients with PD. However, in accordance with our results, Chang et al. [41] found a positive correlation between tongue pressure and the MNA score in a group of community-dwelling adults aged ≥ 65 years. Finally, the presence of a significant association between tongue strength and BMI is in line with the findings of Hara et al. [38] who analyzed a group of 785 healthy individuals.

The association between reduced tongue pressures and swallowing performance during meals or nutritional risk has several implications for clinical practice. Firstly, in patients with PD and dysphagia that exhibit low tongue pressures, a careful monitoring of nutritional status is advisable. With regards to meals, it is known that meal-related fatigue significantly impacts tongue pressures, especially in older people [42]. Therefore, those patients that already have reduced tongue pressure before meals may experience higher fatigue, thus, impacting on swallowing safety and efficiency during meals. In these patients, mealtime management may be improved by selecting foods that require less tongue strength (f.i. considering not only texture, but also hardness or stickiness) or revise meals schedule within the day in favor to smaller but more frequent meals. Consequently, integrating tongue pressure measurements in clinical swallow examination may give clinicians additional information on swallowing function in everyday life. Finally, the results of the study suggest a potential role for lingual resistance training programs in the treatment of dysphagia in PD within the context of a proactive approach [43]. Plaza & Busanello-Stella conducted a randomized controlled study on 60 patients with PD and demonstrated the efficacy of a tongue strengthening training in adjunct to conventional swallow therapy in improving tongue strength and the activation of suprahyoid muscles [44]. However, its impact on swallowing function was not investigated and was found to be highly heterogeneous in another study [45]. Thus, the area requires further investigation.

### Study Limitations

While this study analyzed the association between tongue strength and endurance and mastication, meal and diet for the first time, it has several limitations. First, the number of enrolled patients is quite low and did not reach the targeted sample size. Although the size of the enrolled cohort is in line with previous studies, the loss of statistical power requires the results to be viewed as preliminary and consequently should be interpreted with caution. Second, the study sample consists of a majority of males (75%) and elderly patients (75% aged ≥ 70). It is known that tongue pressures are influenced both by sex and age [46]. However, the predominance of elderlies and males reflects the epidemiology of PD both worldwide and in Italy [1, 47–49]. Additionally, the MNA is commonly used to assess malnutrition risk in neurodegenerative diseases. However, it provides a screening of malnutrition, but not its diagnosis. Therefore, a complete nutritional assessment is advisable in future studies. Finally, information on dental status of recruited patients was not investigated, such as the number of teeth, the presence of fixed or removable prostheses, and their material composition. As these factors can significantly affect mastication and, consequently, oral phase efficiency, their influence on the results of the study cannot be determined and warrants further research.

## Conclusion

In patients with PD, reduced tongue strength and endurance seem to be significantly associated with the worsening of oral phase efficiency and swallowing safety during meal, the restriction of oral diet, and an increases risk of malnutrition. This is of clinical relevance since these tongue parameters may be easily assessed in clinical practice. Additionally, lingual strengthening training is an existing treatment approach for dysphagia and may represent a possibility to improve swallowing function in PD, although the literature is currently scarce. The high prevalence of swallowing, meal, mastication, and nutrition abnormalities found in the present study highlight the importance of a multidimensional assessment extending beyond a swallow study in patients with PD.

## Data Availability

The data that support the findings of this study are not openly available due to reasons of sensitivity and are available from the corresponding author upon reasonable request.
